# Supplementary material for: Redirecting Imipramine against Bluetongue Virus Infection: Insights from a Genome-wide Haploid Screening Study
Source: Pathogens. 2022 May 22;11(5):602. doi: 10.3390/pathogens11050602 (PMC9144988; doi:10.3390/pathogens11050602)
Supplement: Supplementary file 1 [file pathogens-11-00602-s001.zip › pathogens-1705060-supplementary.pdf]

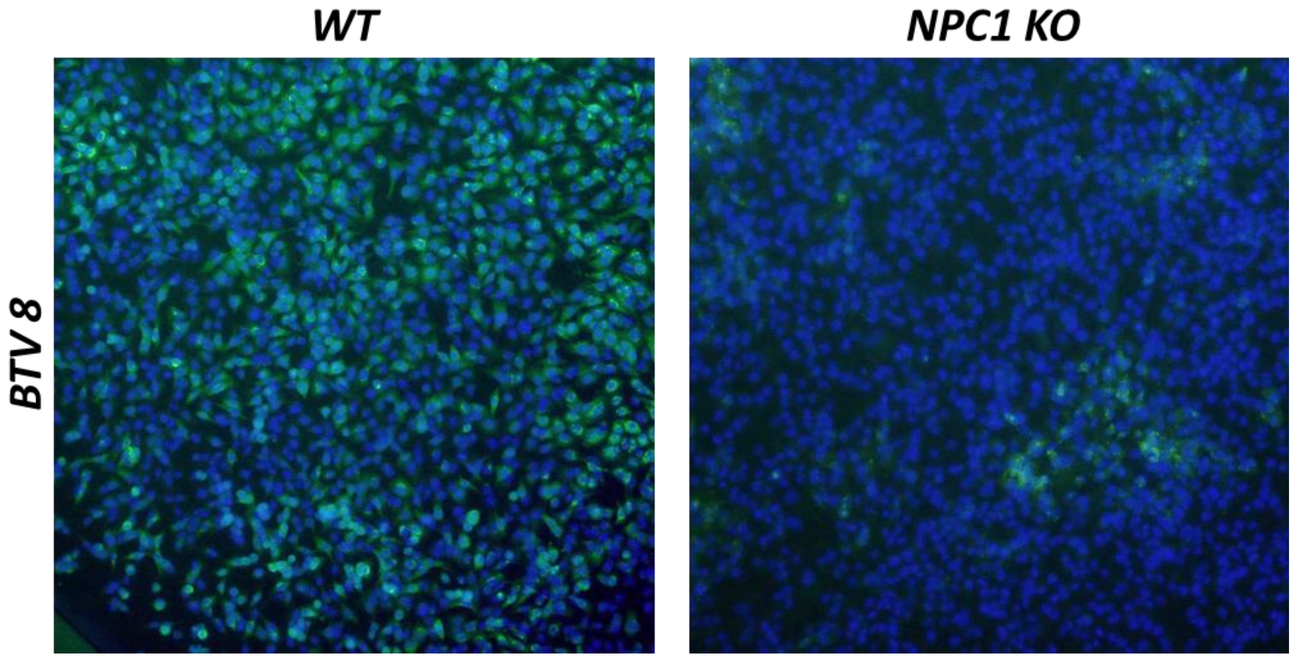

Supplementary Figure S1:
A549 cells deficient for NPC1 (NPC1 KO) and the control cells (Non targeting control) were infected with BTV-8 and stained with a (BTV) FITC conjugate MAb to determine infectivity.

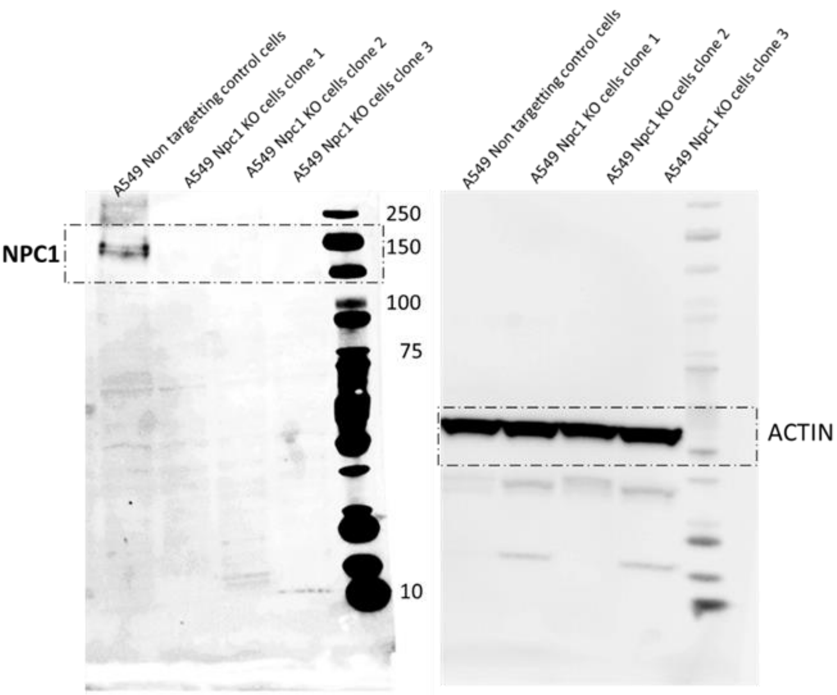

Supplementary Figure S2 :
Western blot analysis of NPC1 protein levels in A549 control cells (Non targeting control) and NPC1 knock out cells [18, 2 and 3]. Actin was used as a loading control.
:

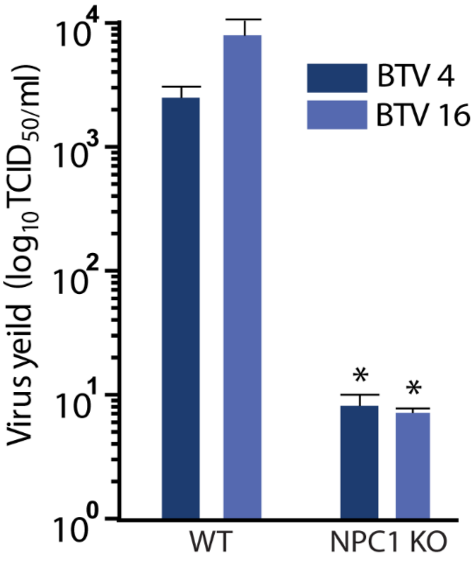

Supplementary Figure S3 : Total virus yield from cell culture supernatant of WT control cells and NPC1 KO cells infected with either BTV 4 or BTV 16. (n= 3, mean ± SD, Student's t test *P < 0.01)

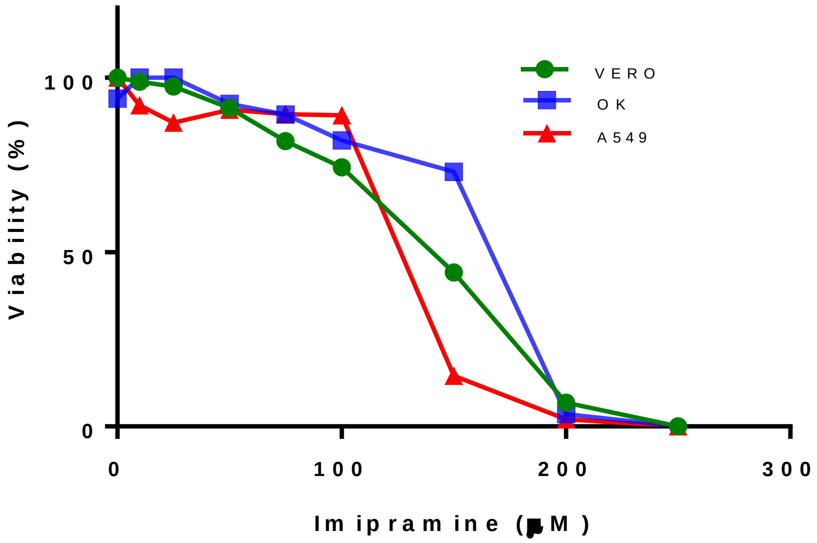

1.

Supplementary Figure S4
Cell viability of Vero, A549 and MDOK cells was measured by cell titer glo assay. Cells were treated with varying concentrations of imipramine. After 48h post treatment cell viability was measured and plotted as percentage viability compared to the DMSO treated control.
